# WGCNA based identification of hub genes associated with cold response and development in *Apis mellifera* metamorphic pupae

**DOI:** 10.3389/fphys.2023.1169301

**Published:** 2023-05-04

**Authors:** Chenyu Zhu, Xinjian Xu, Shujing Zhou, Bingfeng Zhou, Yiming Liu, Hongzhi Xu, Yuanmingyue Tian, Xiangjie Zhu

**Affiliations:** ^1^ College of Animal Science (College of Bee Science), Fujian Agriculture and Forestry University, Fuzhou, China; ^2^ Honeybee Research Institute, Fujian Agriculture and Forestry University, Fuzhou, China

**Keywords:** honeybee, cold response, development, pupae, WGCNA, hub genes

## Abstract

Honeybee is a crucial pollinator in nature, and plays an indispensable role in both agricultural production and scientific research. In recent decades, honeybee was challenged with health problems by biotic and abiotic stresses. As a key ecological factor, temperature has been proved to have an impact on the survival and production efficiency of honeybees. Previous studies have demonstrated that low temperature stress can affect honeybee pupation and shorten adult longevity. However, the molecular mechanism underlying the effects of low temperatures on honeybee growth and development during their developmental period remain poorly understood. In this paper, the weighted gene co-expression analysis (WGCNA) was employed to explore the molecular mechanisms underpinnings of honeybees’ respond to low temperatures (20°C) during four distinct developmental stages: large-larvae, prepupae, early-pupae and mid-pupae. Through an extensive transcriptome analysis, thirteen gene co-expression modules were identified and analyzed in relation to honeybee development and stress responses. The darkorange module was found to be associated with low temperature stress, with its genes primarily involved in autophagy-animal, endocytosis and MAPK signaling pathways. Four hub genes were identified within this module, namely, loc726497, loc409791, loc410923, and loc550857, which may contribute to honeybee resistance to low temperature and provide insight into the underlying mechanism. The gene expression patterns of grey60 and black modules were found to correspond to the developmental stages of prepupae and early-pupae, respectively, with the hub genes loc409494, loc725756, loc552457, loc726158, *Ip3k* and *Lcch3* in grey60 module likely involved in brain development, and the hub genes loc410555 in black module potentially related to exoskeleton development. The brown module genes exhibited a distinct pattern of overexpression in mid-pupae specimens, with genes primarily enriched in oxidative phosphorylation, citrate cycle and other pathways, which may be related to the formation of bee flying muscle. No related gene expression module was found for mature larvae stage. These findings provide valuable insights into the developmental process of honeybees at molecular level during the capped brood stage.

## 1 Introduction

The growth and development of insects are significantly influenced by environmental factors, with temperature being a key variable. Challenged with biotic and abiotic stresses, honeybee health problems arouse much concerns, it is increasingly important to investigate the impact of temperature on insect development. Honeybees are an example of insect that undergo complete metamorphosis, with the egg, larva and pupa (collectively referred to as brood), and adult stages. Stenothermy is a key feature of the honeybee’s brood development, as the temperature range required for the growth and development of the capped brood, which includes mature larvae and pupae, is extremely narrow. The ideal temperature for this stage of development is 35°C, with acceptable temperatures ranging from 29°C to 38°C (Zhu et al., 2006). Any deviation from this temperature range can lead to notable consequences, affecting the brood’s growth and development, disease resistance, stress resistance, and adult worker behavior (Tautz et al., 2003; Groh et al., 2004; Stabentheiner et al., 2021). Our previous research has shown that exposure to low temperature stress during the capped brood stage can result in honeybee mortality, longer developmental periods, shorter longevity (Wang et al., 2016), and potential wing vein variations in adult bees (Zhu et al., 2018). However, the underlying mechanism of how low temperature affects honeybee growth and development remains unclear.

Based on our team’s previous research results, it has been established that honeybees of different stages exhibit varying sensitivity to temperature at 20°C. The developmental stages of honeybees have been defined using Wang’s method, which includes L0 (the last day of mature larvae), PP3 (second day of prepupae), P6 (third day of pupae) and P9 (sixth day of pupae). The letters denote the brood state, while the numbers indicate the number of days following sealing. Among these stages, honeybees in the PP3 stage were found to be the most sensitive to low temperature, followed by P6, P9, and L0. Transcriptome data indicated that low temperature of 20°C might affect the signal transduction of ecdysone through phosphorylation of FoxO pathway genes, affecting the pupation process of honeybee during PP3 (Yao et al., 2020). The transcriptome results of honeybees PP3, P6, and P9 treated at 20°C showed that there were many different genes in autophagy-animal pathway, and honeybee pupae may resist the effects of low temperature stress by regulating autophagy-animal (Liu et al., 2022). At present, the study of transcriptome is limited to comparing differential expression genes between sample groups. WGCNA can reflect the co-expression and interaction of various genes (Zhang and Horvath, 2005). As a new systems biology analysis method, it can be used to identify highly coordinated gene sets, mine relevant specific modules, and screen hub genes.

In this research, the method of WGCNA was employed to analyze the transcriptome samples of L0, PP3, P6, and P9 that were subjected to low temperature stress. The objective was to explore the hub genes at each developmental stage and gain insights into the developmental status of honeybees during these stages. Additionally, the results of this study focus on the hub genes affected by low temperature stress, which can explain the resistance mechanism of honeybee to low temperature stress on the level of gene expression. The findings of this study could offer novel perspectives on the mechanism of cold stress resistance in stenothermic insects.

## 2 Materials and methods

### 2.1 Sample acquisition

During the period of colony propagation from April to June, honeybee samples were collected from colonies. To obtain eggs of the same age, the queen was restricted to lay eggs on an empty comb. After 8 days, when the larvae were old enough to be capped, the capped broods were obtained within a span of 2 h. These capped broods were then incubated in incubators with a constant temperature and relative humidity [Stekai Instruments (Shanghai) Co., Ltd., CTHI-250B, 35°C ± 0.2°C and RH 75%] for 0, 3, 6 and 9 days (Wang et al., 2016). The control group continued to kept under the same conditions for 4 h, while the low temperature treatment groups of AmC-0, AmC-3, AmC-6 and AmC-9 were transferred to an incubator to treated with 20°C ± 0.5°C and RH 75% for 4 h, which were recorded as AmT-0, AmT-3, AmT-6 and AmT-9 respectively. The samples were immediately frozen in liquid nitrogen and transferred to a cryogenic refrigerator at −80°C for storage. There were at least 10 honeybees in each repeated group. Samples were taken from three different colonies for three biological replicates and resulting in a total of 24 group samples in this experiment.

### 2.2 High throughput sequencing and data quality control

The construction of sample library and high-throughput sequencing were entrusted to Guangzhou Omicshare Biotechnology Co., Ltd. using Illumina HiSeq™ 4000 platform. The offline data was filtered using fastp to eliminate the reads containing adapters, the reads containing more than 10% unrecognized bases, the reads with all adenine content, and the reads with a quality value of Q ≤ 20 accounting for more than 50% of the whole read to get clean reads (Chen et al., 2018). The resulting clean reads were then compared to the genomic Amel_HAv3.1 of the western honeybee (NCBI Assembly: GCF_003254395.2). The original data has been uploaded to NCBI’s SRA database with acquisition numbers SRR15258477-SRR15258487, SRR15258489-SRR15258493, SRR15258497-SRR15258498.

### 2.3 Construction of weight co-expression network

The gene co-expression network was constructed using the WGCNA (v1.47) package in R (Langfelder and Horvath, 2008). The FPKM values of 9273 genes were used to construct the gene co-expression network. A total of 24 samples were included in this study, and the optimal soft threshold was selected. The soft threshold selected in this experiment was 11. TOM Type is unsigned, miniMouduleSize = 50, mergeCutHeight = 0.2. Keep the default Settings for other parameters.

### 2.4 Module identification and specific module selection

A gene cluster tree was constructed according to the correlation between gene expression levels, and gene modules were divided according to the clustering relationship between genes. Genes with similar expression patterns were classified into the same module, and branches of the cluster tree were cut and distinguished to produce different modules, with each color representing one module. After the preliminary module division, the result of Dynamic Tree Cut of the preliminary module division was obtained. Since some modules were very similar, we combined the modules with similar expression modes according to the similarity of the characteristic values of the modules, and obtain the final module Merged dynamic. Use the module eigenvalues to draw the sample-module expression pattern heat map, which can directly reflect the expression patterns of each module in each sample, so that we can select the hub modules.

### 2.5 Enrichment analysis

The module genes were analyzed by GO functional enrichment and KEGG pathway annotation. Use the omicshare platform for drawing (http://www.omicshare.com/Tools).

### 2.6 Mining of hub genes

Cytoscape_v3.9.1 was used to analyze the gene with module weight value >0.15 (Shannon et al., 2003) for each module, we further mined the hub gene in the key module, and the hub gene could be screened by the “degree (i.e., the number of genes linked to the gene)” obtained by network analysis. Here, we selected the top 30° values in each module as the screening condition for hub gene (Ma et al., 2019).

## 3 Result

### 3.1 Network construction of weighted co-expression genes

**FIGURE 1:**
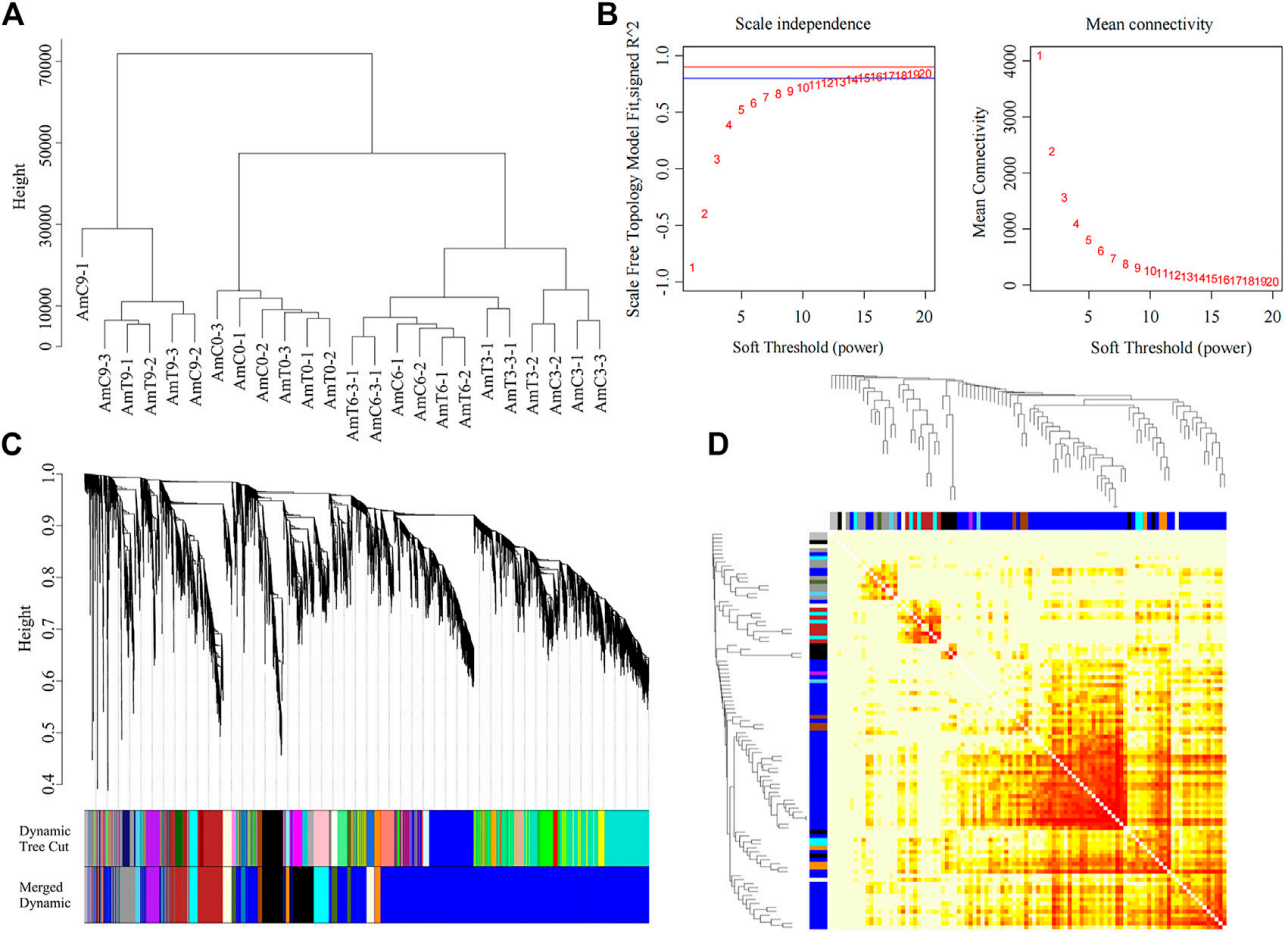
Co-expression network generation in gene level by WGCNA. **(A)** Sample clustering with the FPKM of all genes from twenty-four RNA-seq libraries. **(B)** Soft threshold selection based on the recommended power value. **(C)** The cluster tree of all genes in 13 modules was obtained by heterogeneity. Each vertical line represents a single gene. **(D)** Module gene-related heat map.

Utilizing WGCNA, a network of 9,273 genes was builded. Figure 1A illustrated a strong correlation between gene expression patterns and age. Specifically, all P9 samples during the middle pupal period were clustered together and distinguished from other developmental stages. Furthermore, all the samples of the newly capped larva L0 were gathered together, while the gene expression patterns of the P3 and P6 larvae were more similar, clustering under a large module.

SoftThreshold value was 11, Dynamic Tree Cut was used for the preliminary module division results, and then the modules with similar expression patterns were combined according to the similarity of the module eigenvalues, and the final module Merged dynamic was obtained. Thirteen gene modules with highly similar expression patterns were detected (Figure 1).

### 3.2 Identification of modular genes and selection of specific modules

WGCNA was used to further explore the gene function of each stage, with a mergeCutHeight of 0.2. This analysis resulted in the division of genes into 13 distinct color modules, namely, blue, black, brown, cyan, grey60, lightyellow, darkolivegreen, purple, darkorange, grey, skyblue, and saddleblue. The number of genes in each module were as follows: 5414, 856, 837, 474, 365, 298, 234, 228, 179, 136, 86, 84, and 82, respectively.

**FIGURE 2:**
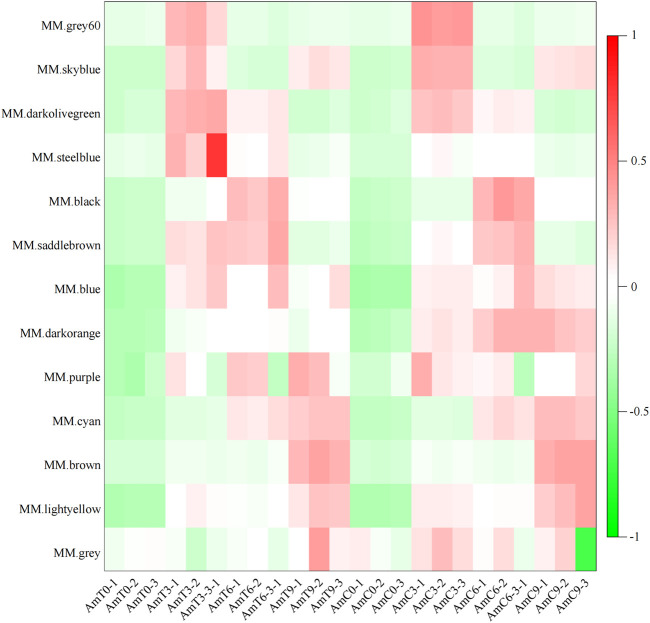
Module-sample gene expression pattern. Abscissa is the sample, ordinate is the module, and the eigenvalues of the module are used to draw the picture.

The expression pattern depicted in Figure 2 reveals that the gene of the grey60 module displayed a relatively high expression level on primarily at the age of 3 days during the capped stage. The gene of black module demonstrated a high level of expression at 6 days of age during the capped stage. The expression level of brown module gene exhibited a high level of expression at 9 days of age during the capped stage. In contrast, the gene expression level of the darkorange module was significantly decreased at 3, 6 and 9 days of age during the capped stage compared to the control group, indicating that the genes in darkorange module were most affected by low temperature stress.

**FIGURE 3:**
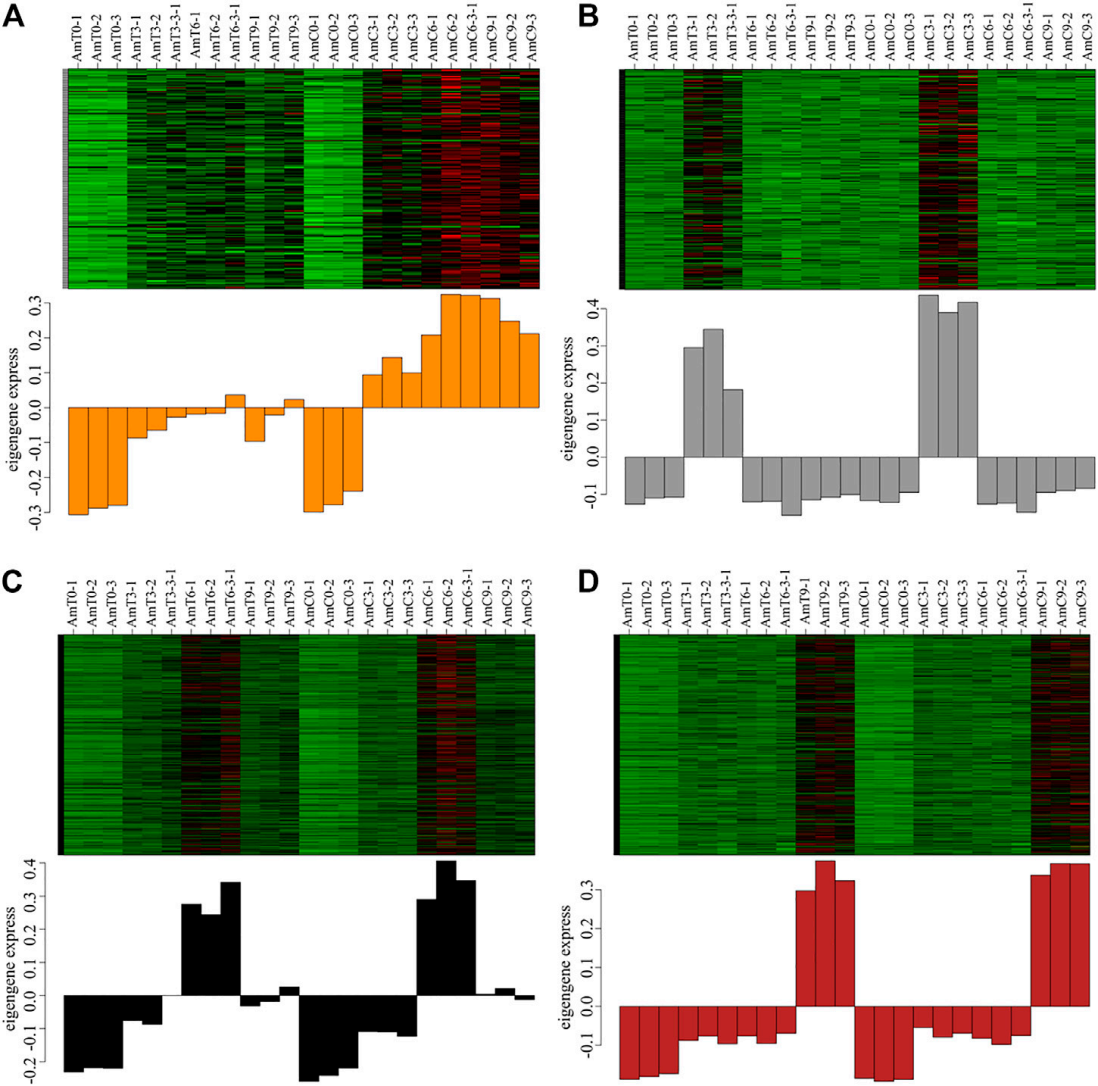
**(A–D)** the expression patterns of darkorange, grey60, black and brown module genes. Red represents high expression and green represents low expression.

The investigation of the module-sample analysis has indicated that the darkorange module gene was associated with temperature stress (Figure 3A). Additionally, grey60, black and brown modules were distinctive to a specific developmental stage or the sample of Figures 3B–D. Specifically, the expression patterns of module genes, grey60, black and brown module genes were highly expressed during the capped stage of 3, 6 and 9 days of age, respectively, which corresponds to the developmental stage of honeybees. As a result, these four modules were deened as feature modules and required undergo further analyze.

### 3.3 Enrichment analysis of module genes

Four modules was selected for enrichment analysis based on their expression pattern, which were significantly altered by age and temperature treatment. The analysis focused on KEGG pathway (*p* < 0.05) and the primary GO item (*p* < 0.01).

**FIGURE 4:**
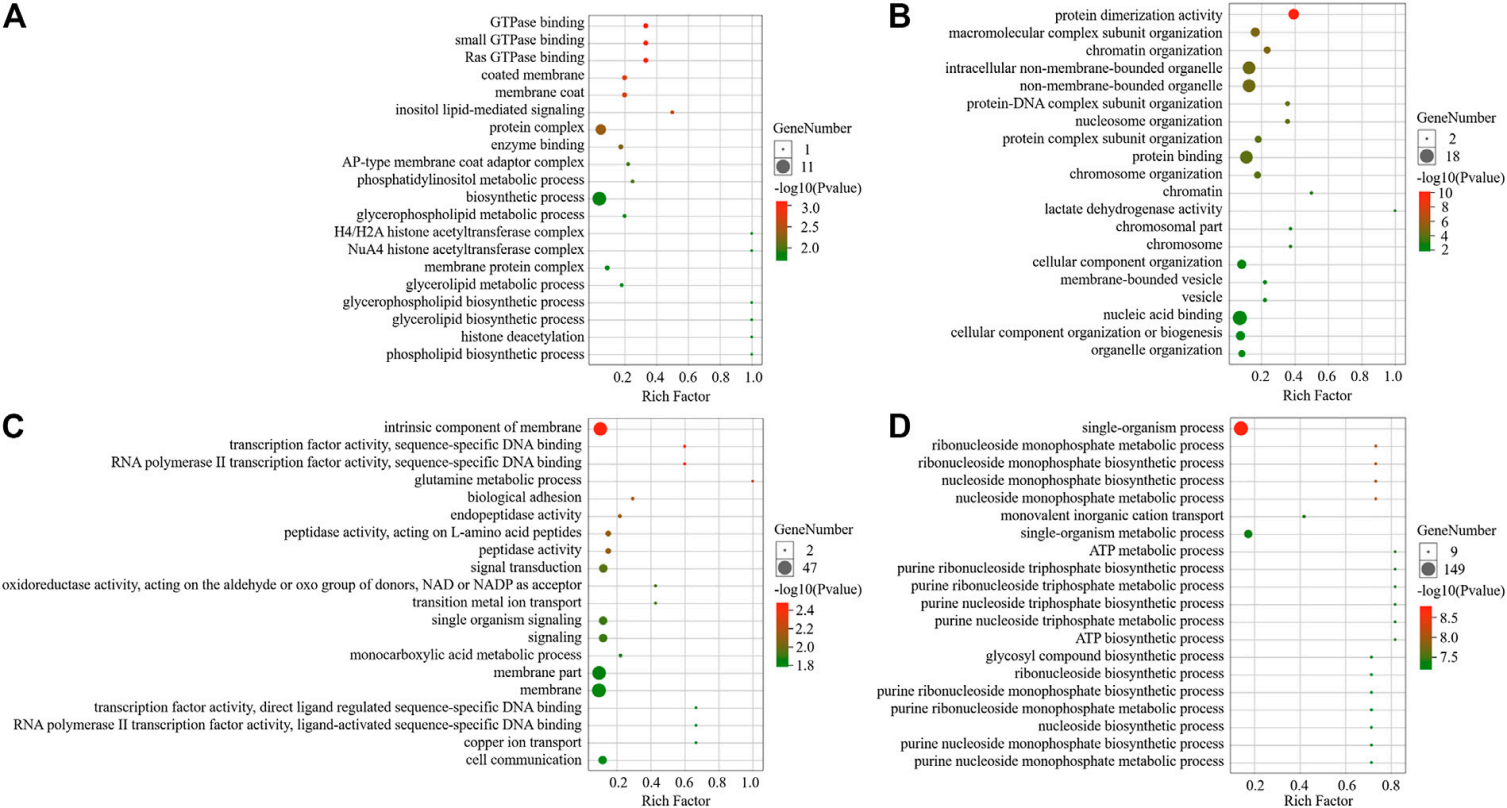
GO enrichment analysis of module. **(A)** Darkorange module enrichment analysis; **(B)** Grey60 module enrichment analysis; **(C)** Black module enrichment analysis; **(D)** Brown module enrichment analysis.

The results of the GO enrichment analysis indicated that the grey60 module gene was strongly correlated with the PP3 stage and its module genes were associated with the biological processes of macromolecular complex subunit organization, chromatin organization, nucleosome organization, protein-DNA complex subunit organization, protein complex subunit organization, chromosome organization and cell component organization. Its cellular components were mainly non-membrane-bound organelle, intracellular non-membrane-bound organelle, and the genes were found to have molecular functions such as protein dimerization activity, protein binding, lactate dehydrogenase activity and nucleic acid binding. The black module genes were found to be highly correlated with P6 periods, and its genes were associated with biological processes such as glutamine metabolism processs and biological adhesion. The main cellular components were the intrinsic component of the membrane, which had functions such as RNA polymerase II transcription factor activity, sequence-specific DNA binding, transcription factor activity, sequence-specific DNA binding, endopeptidase activity and peptidase activity. The brown module genes were found to be highly correlated with P9, and its genes were significantly enriched in biological processes such as single-organism process and single-organism metabolism process. The cellular components were mainly the intrinsic component of membrane and membrane, and they had molecular functions related to oxidoreductase activity, transmembrane transporter activity, transporter activity and other functions. Finally, the darkorange module genes were related to the corresponding low temperature, and its genes were associated with inositol lipid-mediated signaling biological process. Its cellular components consisted of coated membrane, membrane coat and protein complex, with functions such as Ras GTPase binding, small GTPase binding, GTPase binding and enzyme binding (Figure 4).

KEGG pathway analysis showed that grey60 genes were significantly enriched in ECM-receptor interaction, Phenylalanine metabolism and Phagosome pathways. The genes of black module were mainly enriched in Metabolic pathways, Hippo signaling pathway-fly, and Wnt signaling pathway. Brown module pathway genes were mainly enriched in Oxidative phosphorylation, Metabolic pathways, TCA cycle and Glycolysis/the Gluconeogenesis pathway. In terms of the gene expression pattern of darkorange module, compared with the AmC-3, AmC-6, AmC-9, the genes’ expression level of darkorange module were significantly reduced to the corresponding low temperature treatment group. The genes in the module participate in several biological pathways, namely, Hedgehog signaling pathway fly, MAPK signaling pathway-fly, and autophagy-animal (Table 1).

**TABLE 1:** KEGG pathway with significant module differences.

Module	Description	*p*-value
Darkorange	Hedgehog signaling pathway—fly	7.40E-03
	Other types of O-glycan biosynthesis	1.14E-02
	Basal transcription factors	2.11E-02
	Amino sugar and nucleotide sugar metabolism	4.03E-02
	MAPK signaling pathway—fly	4.63E-02
	Autophagy—animal	4.83E-02
Grey60	ECM-receptor interaction	1.80E-03
	Phenylalanine metabolism	1.79E-02
	Selenocompound metabolism	2.95E-02
	Phagosome	3.37E-02
	Glycine, serine and threonine metabolism	4.88E-02
Black	Metabolic pathways	1.20E-03
	Ether lipid metabolism	2.10E-03
	Hippo signaling pathway—fly	1.35E-02
	Hedgehog signaling pathway—fly	2.84E-02
	Wnt signaling pathway	3.82E-02
	Alanine, aspartate and glutamate metabolism	4.33E-02
Brown	Oxidative phosphorylation	4.24E-47
	Metabolic pathways	7.08E-16
	Citrate cycle (TCA cycle)	5.22E-07
	Carbon metabolism	5.77E-05
	Ribosome	3.10E-03
	Pyruvate metabolism	5.40E-03
	Glycolysis/Gluconeogenesis	2.80E-02
	Tyrosine metabolism	2.95E-02

### 3.4 Hub gene identification of module

**FIGURE 5:**
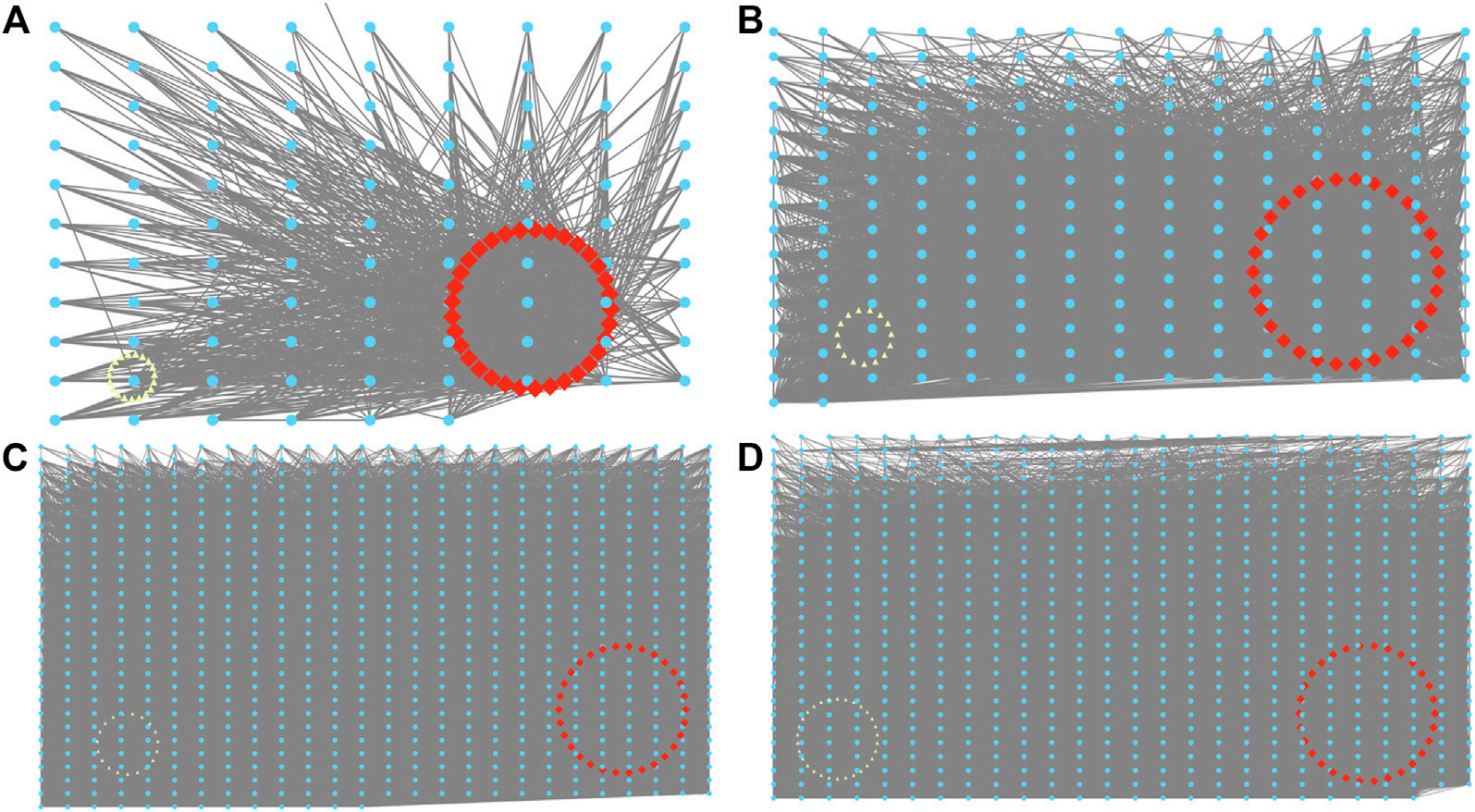
Visualization of the eight co-expression networks by Cytoscape. **(A)** Darkorange. **(B)** Grey60. **(C)** Black. **(D)** Brown. Red is the 30 genes with the highest correlation degree, and yellow is the gene whose correlation degree <5.

By utilizing cytoscape, a network was produced, and degree was obtained from genes with weight value >0.15 in darkorange, grey60, black, and brown modules (Figures 5A–D). The degree was employed as the condition for screening hub genes, leading to the identification of a total of 120 hub genes from the four modules (Table 2).

**TABLE 2:** Module hub genes.

Module	Hub genes
Darkorange	loc410923, loc408401, loc413288, loc550806, loc551976, loc726543, loc410487, loc100578888, loc724660, loc726497, loc413260, loc412770, loc551493, loc107963982, loc413052, loc408373, loc413793, loc413159, loc411812, loc726358, loc725474, loc411163, loc411629, loc726723, loc552297, loc413755, loc550857, loc409791, loc411661, loc411699
Grey60	*CSP6*, loc408538, loc113219298, loc100578780, loc724458, loc100578727, loc412382, loc413749, loc726451, loc409494, loc725756, loc551779, loc408552, loc102655429, loc113218662, loc550870, loc409950, loc552679, loc107965562, loc552457, loc100577163, loc100578084, loc726158, loc726165, loc408572, *Ip3k*, loc550827, LCCH3, loc413207, loc409260
Black	loc413562, loc100578253, loc726051, loc408405, loc551432, loc412795, loc410606, loc724569, loc412354, loc726298, loc408896, loc552257, loc727000, loc102655479, loc408803, loc724613, loc550655, loc724243, loc726529, loc410555, loc102655706, loc552101, loc410427, loc411888, loc102656198, loc551144, loc410649, loc550735, loc102655319, loc550970
Brown	*Eaat-2*, loc411411, loc551866, loc552048, loc107964089, loc725015, loc100577530, loc410557, loc551039, loc409087, loc113218599, loc409236, loc725404, loc411732, loc412862, loc107964828, loc725491, loc413891, loc409292, loc408477, loc409793, loc409931, loc726441, loc409473, loc412890, loc102654955, loc724629, loc408394, *Gat-a*, loc410058

Hub genes of four modules were analyzed. Our analysis revealed that the grey60 genes exhibited high expression levels during the PP3 stage, and the gene with the highest connectivity in this module was *Csp6*, a chemical reactive protein. Similarly, the black module genes displayed high expression levels at 6-day-old capped broods, and its most connected gene was loc413562. Likewise, the brown module genes exhibited high expression levels at 9-day-old capped broods, and *Eaat-2* was identified as the gene with the highest connectivity in its module. Furthermore, the darkorange module genes displayed a decrease in gene expression levels after low temperature treatment at 3, 6 and 9 days old during the capped stage, and the gene with the highest module connectivity was loc410923.

## 4 Discussion

To explore the hub genes of honeybees at various developmental stages and to understand the mechanism of low temperature resistance during the cap development stage, we selected a few representative developmental stages of the honeybee for analysis. This study used WGCNA to analyze the transcriptome data of honeybee brood of L0, PP3, P6 and P9 that had been exposed to 20°C for 4 h, so as to pinpoint hub genes with different developmental patterns and to investigate the hub gene and its molecular mechanism in response to low temperature.

### 4.1 Hub genes in prepupal honeybee development

The PP3 period is the pivotal moment in the development of honeybees, as the transformation from large larvae to pupae takes place, with a variety of physical modifications. PP3 is the time when the honeybee’s mushroom brain experiences the highest level of neuroblast division. As the progenitors of kenyon cells, neuroblasts are indispensable for the assembly and operation of the mushroom body in the subsequent stage (Farris et al., 1999; Strausfeld, 2002). The grey60 module was found to reflect the PP3 correlation in the research results, so further enrichment analysis of the genes in the module was conducted to explore the role of the hub gene in the developmental stage of the module. Among the 30 hub genes studied, the pathway with the most enriched genes is the metabolic pathway, which contains five genes: loc409494, loc725756, loc552457, loc726158 and *Ip3k* (inositol 1,4,5-triphosphate kinase). *Ip3k* is a core enzyme in the IP_3_-mediated inositol phospholipid transduction cascade. It can phosphorylate inositol-1 5-trisphosphate Inositol-1,4,5-trisphosphate (IP_3_) into inositol-1,3,4,5-trisphosphate (IP_4_), thus regulating the concentration of Ca^2+^ (Irvine, 1991). The signal resulting from the modification of Ca^2+^ concentration can modulate neuronal and sensory signals, as well as other neural activities, and is also involved in neural plasticity, which is vital for many brain functions (Mailleux et al., 1991; Berridge, 1993; Bootman et al., 2001). Studies have indicated that honeybee *Ip3k*, a type A variant of *Ip3k* mainly located in the brain area, has been found to be connected to differences in honeybee behavior when its expression level is changed (Kucharski and Maleszka, 2002). The marked high-level expression of *Ip3k* could be linked to the development of brain and subsequent behavior in adults. In addition, the hub gene *Lcch3*, an important subunit of γ-aminobutyric acid (GABA) gated ion channels (Ménard et al., 2018), showed a high expression during PP3 implies that significant modifications are taking place within the bee brain.

### 4.2 Hub genes in midterm pupal honeybee development

The P6 stage may be related to the hardening and coloration of the exoskeleton (stratum corneum) of honeybees. Our results identified a hub gene insulin-like peptide homologue, insulin-like growth factor (IGF) in P6 stage (loc410555). IGFs, evolutionarily analog to relaxin in metazoans, involved in multiple physiological processes including promoting body growth during development by activating a receptor tyrosine kinase IGF receptor (Mizoguchi and Okamoto, 2013).

The gene loc410555 has also shown linked to the concentration of honeybee molting hormone, an important role in regulating ecdysone in insects (Sarraf-Zadeh et al., 2013; Lee et al., 2018). Prior studies showed that the titer of honeybee molting hormone peaked during the P6 stage (Pinto et al., 2002), and the molting hormone is responsible for the molting process of honeybees, sending out pulses that cause the exoskeleton to be renewed and hardened, taking on a blackened hue. There is a close correlation between exoskeleton hardening and the creation of structural proteins in honeybees (Soares et al., 2013). Besides, Hormones regulate cell metabolism mainly via controlling the activity of protein kinases and protein phosphatases (Pearson et al., 2001), such as the serine threonine protein kinase (loc726051, loc410427) and tyrosine-protein kinase (loc410649) expressed in the P6 stage, which involved in pathways controlling organogenesis, cell differentiation, cell proliferation and cell death in insects (Lewis et al., 1998).

During this period, we hypothesize that bees undergo a process of molting which leads to the tissue remodeling, organogenesis, melanization, hardening of the new cuticle. Our experiments have demonstrated that when bees are exposed to low temperatures during the P6 period, their backplane color darkens, and blackening of adult bees becomes more evident (Wang, 2016).

### 4.3 Hub genes in late pupal honeybee development

Of utmost importance during the P9 phase is the formation of honeybee flight muscle. The P9 period of the honeybee has progressed to its late pupal stage, and its physical form is comparable to that of a normal adult bee. It was observed the number of flight muscle myosome in honeybees, from late pupa to adult maturity, had augmented by 12 times (Herold, 1965). GO enrichment results indicated that the genes found in the brown modules were involved in biological processes such as oxidoreductase activity and transmembrane transporter activity. Results from KEGG analysis showed that these genes were significantly enriched in metabolic pathways associated with ATP, including oxidative phosphorylation and TCA cycle, which are closely linked to the production of ATP in the body (Bonora et al., 2012; Mookerjee et al., 2018). Eight of the 30 hub genes of this module have been identified as being enriched in the Oxidative phosphorylation pathway, NADH dehydrogenase (loc411411, loc551866loc408477, loc409793, loc409473), ATP synthase (loc410557, loc102654955) and NADH-ubiquinone oxidoreductase (loc413891), suggesting it need a great amount of energy at P9 stage (Grivennikova et al., 2020). Our results indicate that the energy activity in P9 period could be associated with the formation or advancement of bee flying muscles. It is speculated that the energy produced by mature mitochondria at the age of P9 is utilized by honeybees to facilitate the gradual maturation of their flight muscles.

### 4.4 Hub genes in pupal honeybee response to low temperature

The honeybee pupae are stenotherm that adapted to stable nest temperature sustained by the colony worker bees. Whether the brood honeybee have abilities to resist lower temperature deviated from its optimal ones remain little known. Our data found that brood honeybee may respond to low temperature by regulating genes in autophagy pathway. Hug genes in the darkorange module revealed significant differences in expression patterns at PP3, P6 and P9 when compared to the control group post low temperature treatment. The KEGG enrichment results indicated that the differential genes were implicated in autophagy-animal and endocytosis (loc726497, loc409791, loc410923), suggesting that low temperature impacted the development of honeybees by changing autophagy process. The loc726497, homologous to *Atg2* in *Drosophila melanogaster*, was capable of regulating autophagy by transporting lipids to start autophagy biogenesis (Valverde et al., 2019). The loc409791, the homology of *Pka-C1* in *Drosophila* and encoding a cAMP-dependent protein kinase catalytic subunit (Cassar et al., 2018; Skoulakis et al., 1993), plays important roles in autophagy during development and resistance to biotic stress (Grisan et al., 2021; Yu and Rollins, 2022).

The homologous gene of loc550857 in *Drosophila* is described as *Drk*, which may participate in the formation of mushroom body synapse and affect the memory of *Drosophila* (Kotoula et al., 2017), suggesting its shifted expression level affecting mushroom development and brain function post low temperature. The homologous gene of loc550857 encodes protein enhancer of sevenless 2B, E (sev)2B, a key adapter protein in the Ras/MAPK signaling pathway which has been reported to be involved in innate immunity in *Artemia sinica* response to bacterial challenge (Xia et al., 2019), suggesting it involved in immune response to low temperature stress in pupal development. The gene loc550857 is also enriched in FoxO signaling pathway, which responded to low temperature stress by regulating the growth and development of honeybees (Liu, 2022).

## Data Availability

The datasets presented in this study can be found in online repositories. The names of the repository/repositories and accession number(s) can be found below: NCBI’s SRA database (acquisition number: SRR15258477-SRR15258487, SRR15258489-SRR15258493, SRR15258497-SRR15258498).
